# A high-throughput pipeline for scalable kit-free RNA extraction

**DOI:** 10.1038/s41598-021-02742-w

**Published:** 2021-12-01

**Authors:** Ping Han, Maybelle K. Go, Jeng Yeong Chow, Bo Xue, Yan Ping Lim, Michael A. Crone, Marko Storch, Paul S. Freemont, Wen Shan Yew

**Affiliations:** 1 Synthetic Biology for Clinical and Technological Innovation, National University of Singapore, 28 Medical Drive, Singapore, 117456 Singapore; 2 Synthetic Biology Translational Research Programme, Yong Loo Lin School of Medicine, National University of Singapore, 14 Medical Drive, Singapore, 117599 Singapore; 3 Department of Biochemistry, Yong Loo Lin School of Medicine, National University of Singapore, 8 Medical Drive, Singapore, 117597 Singapore; 4 Section of Structural and Synthetic Biology, Department of Infectious Disease, Faculty of Medicine, Imperial College London, Exhibition Road, South Kensington, London, SW7 2AZ UK; 5 London Biofoundry, Translation and Innovation Hub, Imperial College White City Campus, London, W12 0BZ UK; 6 UK Dementia Research Institute Centre for Care Research and Technology, Based at Imperial College London and the, University of Surrey, London, UK

**Keywords:** Laboratory techniques and procedures, High-throughput screening, Assay systems, Infectious-disease diagnostics

## Abstract

An overreliance on commercial, kit-based RNA extraction in the molecular diagnoses of infectious disease presents a challenge in the event of supply chain disruptions and can potentially hinder testing capacity in times of need. In this study, we adapted a well-established, robust TRIzol-based RNA extraction protocol into a high-throughput format through miniaturization and automation. The workflow was validated by RT-qPCR assay for SARS-CoV-2 detection to illustrate its scalability without interference to downstream diagnostic sensitivity and accuracy. This semi-automated, kit-free approach offers a versatile alternative to prevailing integrated solid-phase RNA extraction proprietary systems, with the added advantage of improved cost-effectiveness for high volume acquisition of quality RNA whether for use in clinical diagnoses or for diverse molecular applications.

## Introduction

Rapid and accurate diagnosis of infectious diseases is invaluable for early therapeutic intervention and effective treatment and is critical for infection control that can have serious repercussions to public health outcomes. Serological and molecular assays are fast superseding cell culture-based methods as timely diagnostic tools in infection surveillance. Due to its high sensitivity of detection, specificity for causative agents, broad dynamic range, and its speed and ease of use, quantitative polymerase chain reaction (qPCR) has become the gold standard procedure in clinical diagnosis, particularly in the diagnosis of viral diseases, such as the arboviruses, hepatitis viruses and HIV^1^. Advanced sequencing technology that expeditiously provides whole-genome sequences of pathogens has allowed PCR-based platforms to be a nimble tool in diagnosing emerging novel infections. The most recent and high-profile use of PCR assays is the molecular identification of SARS-CoV-2 for rapid laboratory diagnosis of Covid-19, a pandemic that has overwhelmed public healthcare globally.

Typically, a clinical diagnostic approach involves viral RNA extraction from human biological samples followed by PCR assay targeting virus-specific nucleic acid. Viral RNA is routinely extracted using commercially available solid-phase RNA extraction kits that are often supplied by a handful of major manufacturers who vertically integrate these kits with their proprietary instruments and essential plastic wares, leaving little or no room for generic suppliers^2^. While undoubtedly streamlined for user convenience, interruption in the supply of any of the components in the integrated system due to limited manufacturing capacity or surge demand can greatly hamper diagnostic capabilities as epitomised by the acute global shortage of kits during the Covid-19 pandemic^3^. Apart from supply chain strain, kit-based approaches also present another challenge—the affordability of the kits and their dedicated instruments, which can be prohibitive to low and low-middle income countries in South Asia, South America and Africa.

To ensure that mass testing in future epidemics is not compromised by the unavailability and/or unaffordability of RNA extraction kits, a robust alternative for RNA extraction that guarantees a stable and scalable supply at low cost while maintaining diagnostic sensitivity and specificity, is acutely needed. Extremely versatile and robust, TRIzol-based reagents have widely been used for isolating high quality RNA from a variety of biological samples including cells, tissues, serum, blood and microbes. The powerful protein denaturant effectively stabilizes RNA and inactivates RNases and infectious agents, allowing the recovery of RNA from very small amount of input samples. However, RNA isolation from a high volume of samples in the conventional 1.5-mL microcentrifuge tube format is incompatible with high-throughput applications. Here we demonstrate that a well-established, TRIzol-based, non-kit RNA extraction protocol can be adapted into a semi-automated, high-throughput format using equipment readily available in research and hospital laboratories. Moreover, recent establishment of Biofoundries around the world, with their versatile and scalable automation pipelines, is poised to lend firm infrastructural support to our approach^4^. Nonetheless, even where automated liquid handling capabilities are lacking, the adapted protocol for a 96-well format can still be carried out manually using a multi-channel pipette, albeit at a slower pace yet remaining cost-effective for acquisition of RNA from large numbers of samples.

## Results and discussion

**Figure 1:**
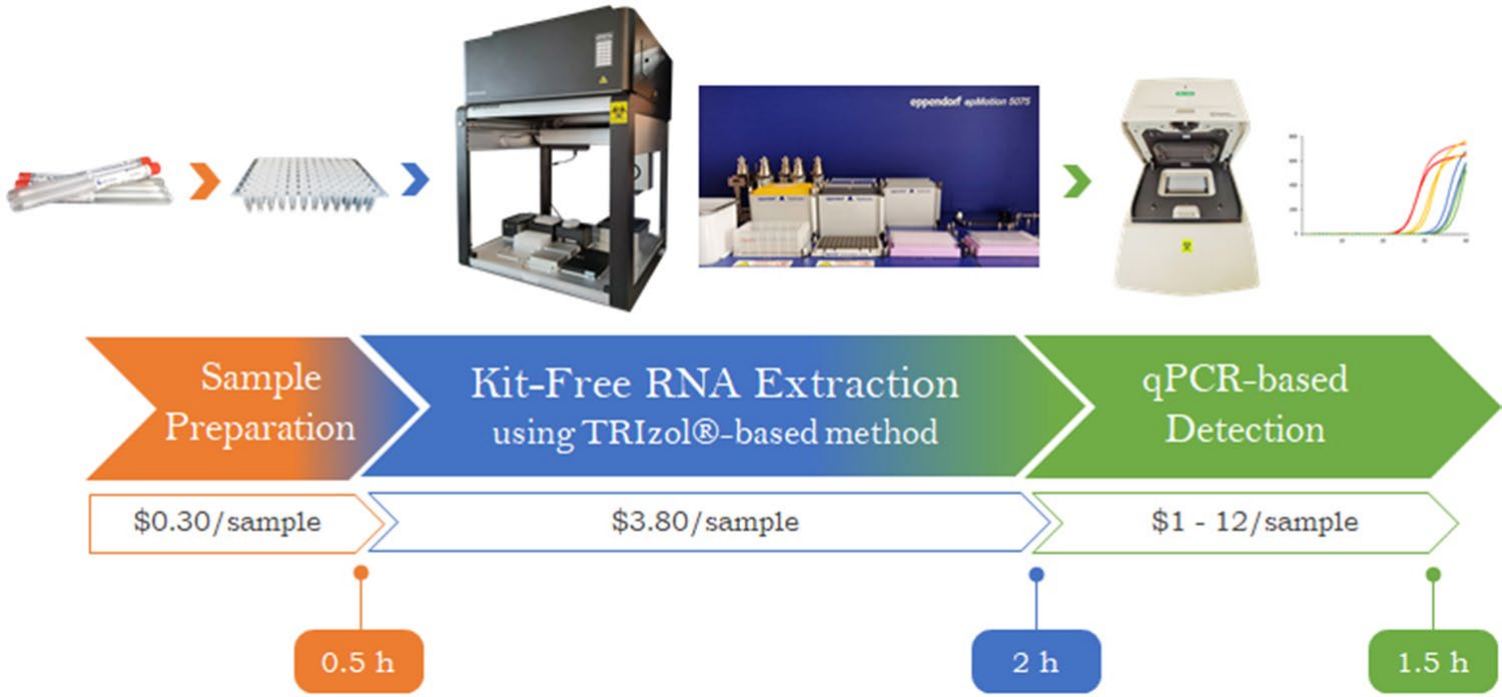
Scalable high-throughput kit-free RNA extraction workflow. Biological samples are manually transferred into a 96-well PCR plate and total RNA extraction was carried out by automated liquid handlers using a modified TRIzol-based method. Manual intervention is required for plate centrifugation. Viral diagnostic analyses is performed on extracted RNA via qPCR-based detection. A complete run of 96 samples takes 4 h. The cost (USD) per sample was calculated based on actual local prices and includes laboratory consumables such as tips and reagents but excludes the cost of instruments.

The TRIzol method, while widely proven effective in RNA extraction, poses challenges to adaptation for high-throughput processing performed on automated liquid handling instruments due to the multiple steps involved. The need for phase separation, precipitation by centrifugation and potential phenol carryover further complicates the workflow. In this study, the conventional protocol was adapted to a 96-well format with emphasis on miniaturization and reduced human intervention (Fig. 1). To validate that the semi-automated, non-kit extraction method was able to isolate RNA at sufficient yield and quality for sensitive downstream applications, biological samples were spiked with non-infectious, synthetic SARS-CoV-2 virus-like-particles (VLPs) and heat-inactivated 2019 novel coronavirus and the extracted RNA evaluated for SARS-CoV-2 detection via real-time reverse transcription qPCR (RT-qPCR). While prevailing clinical diagnoses of SARS-CoV-2 are mostly conducted on nasopharyngeal or oropharyngeal specimens, several studies have shown that saliva presents a low-cost, non-invasive and reliable alternative for SARS-CoV-2 detection^5–7^. As such, saliva and pharyngeal swabs were used as diagnostic samples in this study due to the ease of self-collection from volunteers, negating the need for skilled personnel for sample collection. Importantly, saliva can be highly viscous and difficult to pipet and was presented as an assessment on the automated liquid handlers’ capability to exact extraction from specimens with varying viscosity. For comparison, analyses were also carried out on oropharyngeal nasal mid-turbinate (OP-NMT) swabs performed by a trained medical personnel. Two laboratory automation platforms were employed, namely, the Opentrons OT-2 lab robot and the Eppendorf epMotion® 5075, both which were equipped with an 8-channel volumetric dispensing arm and a thermal block for 96-well plates.

**Figure 2:**
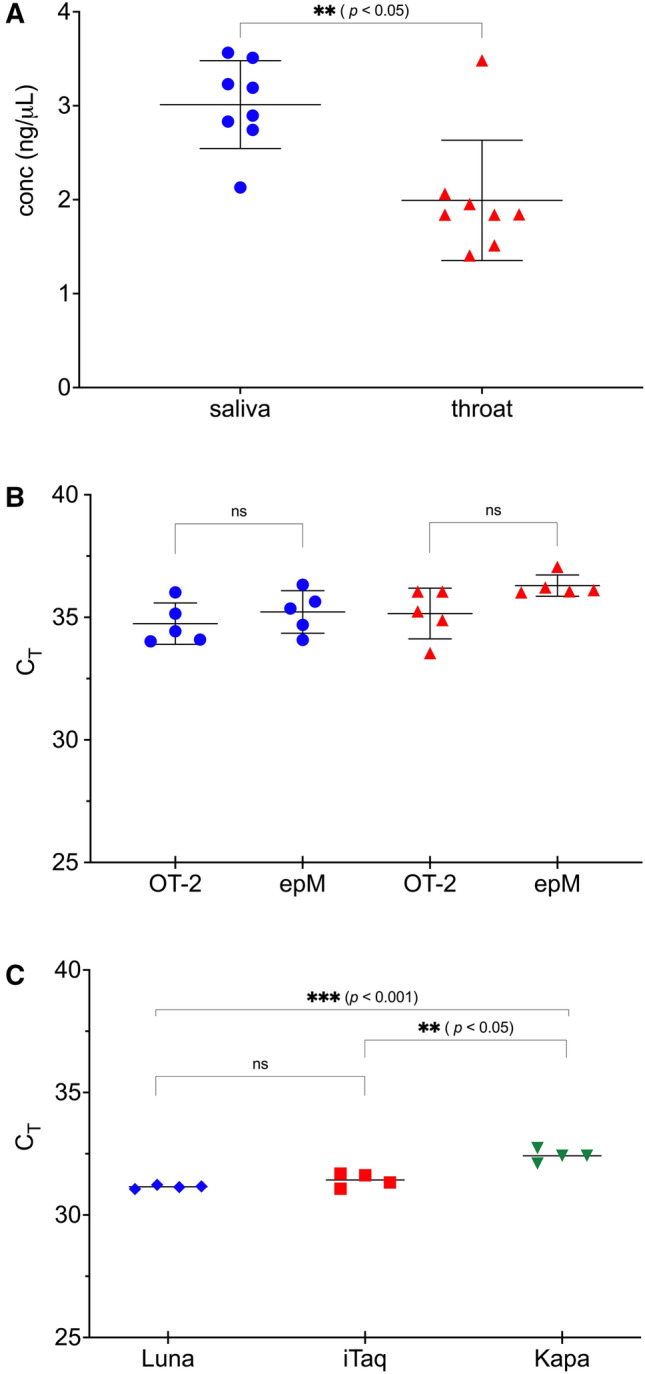
Efficacy of TRIzol RNA isolation from saliva and throat swabs using automated liquid handlers. (**A**) Concentration of RNA of saliva (blue circle) and throat swabs (red triangle) determined using Quant-IT. (**B**) Threshold cycles (C_T_) of one-step RT-qPCR analysis on total RNA extracted from saliva (blue cirlce) and throat swabs (red triangle) using Opentron-2 (OT2) and Eppendorf epMotion (epM). Analysis targeting human Ribonuclease P (RNase P) was performed on five technical replicates. (**C**) Detection of RNase P using multiple qPCR master mixes. Analysis was performed with Luna (NEB; blue diamond), iTaq (Bio-Rad; red square) and KAPA (Kapa Biosystems; green inverted triangle) one-step SYBR RT-qPCR kits. Total RNA was extracted from throat swabs using Eppendorf epMotion. Statistical differences were analysed using unpaired, two-tailed t-test where ns indicates non-significance (*p* > 0.05).

In order to perform RNA isolation in 96-well format, the reaction volume was scaled down proportionally in a 3:1 TRIzol reagent-to-sample ratio, reducing the volume of reagent required by sevenfold to 105-μL, thereby directly lowering the consumable cost per sample extraction. With a concomitant decrease in input sample to a volume of just 45-μL, glycogen was added as a co-precipitant to aid in RNA recovery. In a kit-free extraction method, precipitation and purification of RNA by centrifugation remain ineludible steps, making manual intervention obligatory in the workflow. The addition of glycogen as an inert carrier for nucleic acids was crucial in overcoming the challenge of low centrifugal force when using swing-out rotors for plates. The adapted procedure successfully extracted 1 to 4 ng/μl of RNA from both saliva and throat swabs with the yield slightly higher from saliva (Fig. 2A).

During transfer of the aqueous phase following phase separation, cross-contamination with proteins, lipids, DNA and phenol can occur. Indeed, ensuring high quality of isolated RNA, particularly in a miniaturized format, proved challenging. Phenol carryover was identified in sample extractions using both automated liquid handlers as indicated by a characteristic absorbance maximum at 270 nm. Despite the relatively low purity and quantity, the extracted RNA was tested for its suitability for use in sensitive downstream applications such as real time RT-qPCR. The phenol contamination did not appear to interfere with the efficacy of the RT-qPCR targeted at the ubiquitous human Ribonuclease P (RNase P) from both the saliva and throat (Fig. 2B). To determine the reproducibility in analytical efficiency, the validation was repeated using three commercially available one-step RT-qPCR master mixes (Fig. 2C). The Ct values showed slight variability for the KAPA qPCR mix compared to the Luna and iTaq mix but the results, nonetheless, suggested that the extracted RNA was of sufficient yield and quality for downstream diagnostic application irrespective of the molecular reagent kits.

**Figure 3:**
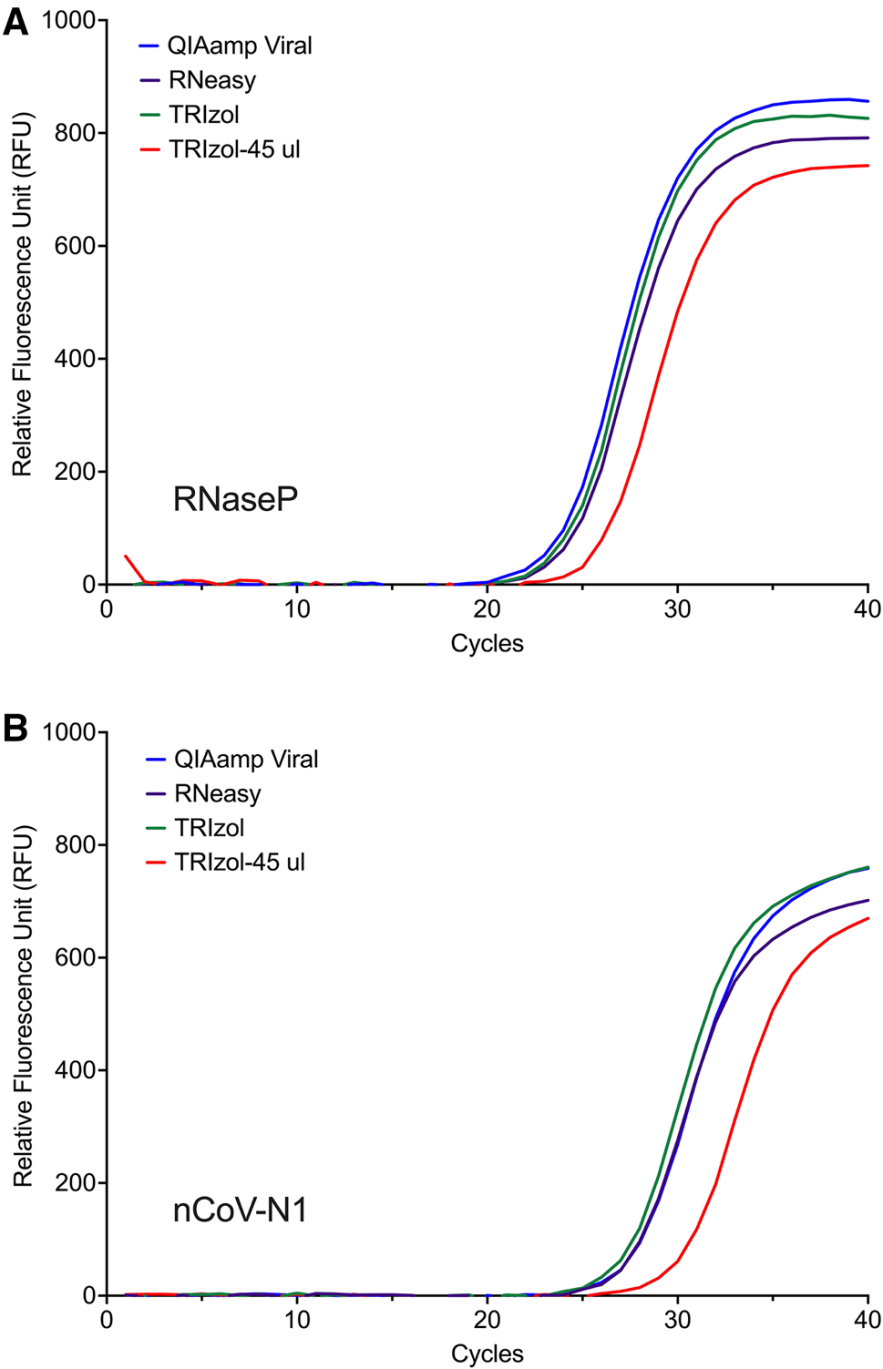
Efficiency of TRIzol RNA extraction relative to commercial kits. RNA was isolated from swab samples spiked with heat inactivated 2019 novel coronavirus (nCoV) using the Qiagen QIAamp Viral RNA kit, Qiagen RNeasy Mini Kit, standard TRIzol-based method performed manually, or semi-automated, 96-well-adapted TRIzol-based protocol by Eppendorf epMotion. SYBR-based amplification curves for (**A**) human Ribonuclease P, and (**B**) SARS-CoV-2 N1 gene.

To assess viral RNA extraction efficiency of the miniaturised, semi-automated TRIzol-based extraction protocol against commercial RNA purification kits, biological samples were spiked with heat-inactivated 2019 novel coronavirus (nCoV) and RNA extracted using the QIAamp Viral RNA Mini Kit specific for viral RNA isolation from body fluids, the RNeasy Mini Kit for generic purification of total RNA from cells as well as the TRIzol-based method performed manually at standard volume. To ensure consistent sample input, 45 ul of biological samples were each spiked with a fixed quantity of 4,500 copies of nCoV, and topped up with DMEM to the varying starting sample volumes recommended for each kit. Kit-extracted RNA was eluted according to manufacturer’s instructions, followed by volume reduction by evaporation to 10 ul to standardize with that used in the miniaturised TRIzol-based method before performing RT-qPCR assay targeted at RNaseP and SARS-CoV-2 N gene. The CDC 2019-nCoV N1 primer set was used as it has been shown to be more sensitive than the N2 or N3 sets, typically generating lower Ct values from positive samples^8,9^. The standard TRIzol-based method gave RNA recovery comparable to the QIAamp Viral RNA kit and RNeasy Mini kit with Ct values of 24.3, 24.1, 24.7 for RNaseP and 27.7, 28.1, 28.1 for nCoV-N1 detection, respectively (Fig. 3A, B). The results were similar to that reported by Won et. al., 2020, indicating that TRIzol was equally effective in extracting RNA compared to commercial available kits^10^. While the miniaturised, 96-well-adapted TRIzol-based extraction yielded adequate RNA to amplify the target genes, the method showed a lower sensitivity as observed by the higher Ct values for RNaseP (Ct = 26.3) and nCoV-N1 (Ct = 30.6).

**Figure 4:**
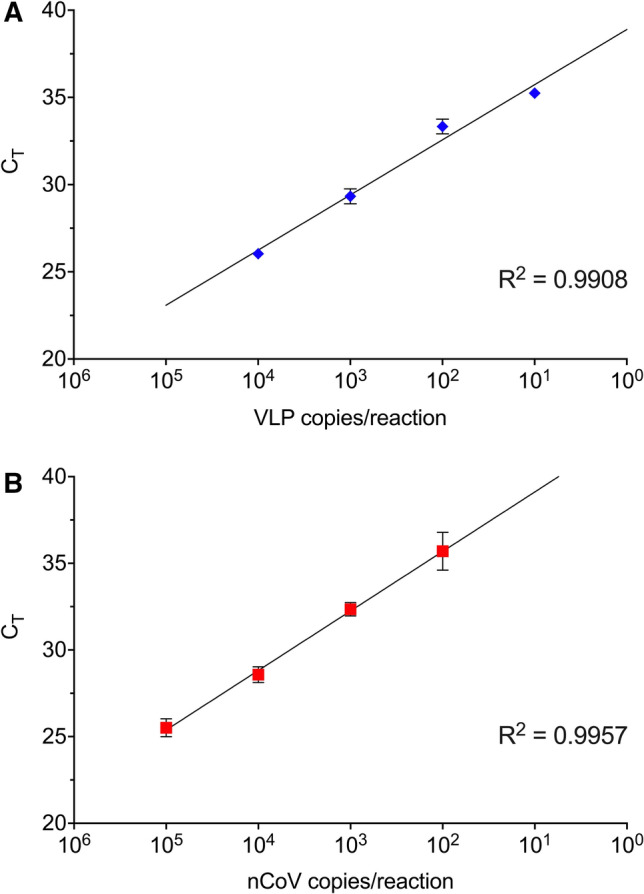
Efficiency of TRIzol RNA extraction from serial-diluted SARS-CoV-2 spiked sample**s.** SYBR one-step RT-qPCR analysis targeting SARS-CoV-2 N gene was performed on DMEM spiked with tenfold serial dilutions of (**A**) SARS-CoV-2 virus-like-particles (VLPs; blue diamond), and (**B**) heat inactivated 2019 novel coronavirus (nCoV; red square). Total RNA was extracted from each concentration using Eppendorf epMotion. Results are shown as mean ± SE of three technical replicates.

The limit of detection of the semi-automated workflow was also examined with tenfold serial dilutions (10^1^–10^5^ copies per reaction) of SARS-CoV-2 VLPs and nCoV. The highest quantified concentration in the standard curve for VLPs was limited to 10^4^ copies per reaction as the available stock concentration was 1700 copies per μL. The extracted RNA showed a detection limit of 10 copies of VLP per reaction, while that of nCoV was 100 copies per reaction, suggesting good efficiency of extraction using the high-throughput platform (Fig. 4).

**Figure 5:**
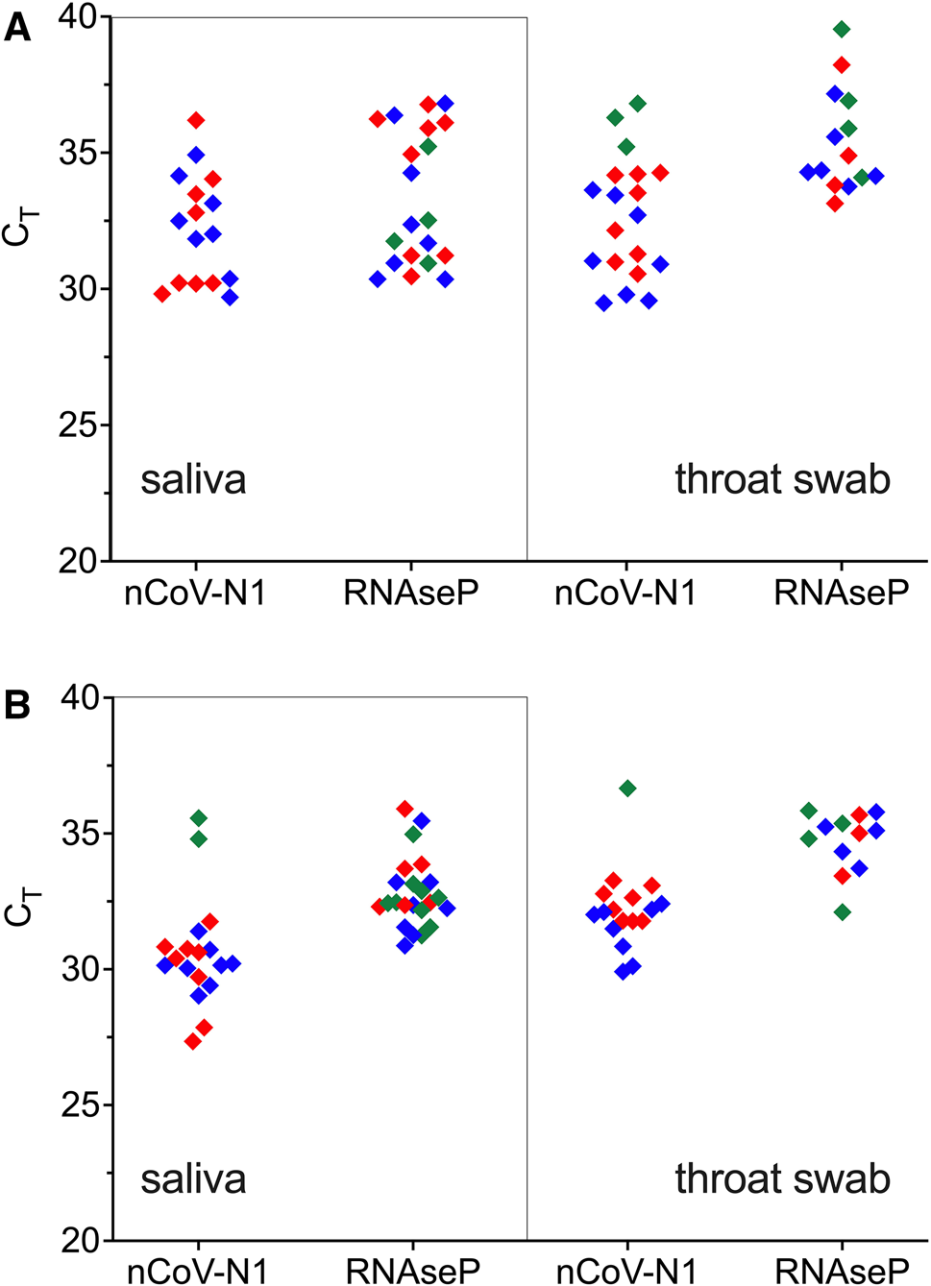
SARS-CoV-2 detection on hypothetical patient samples. Saliva and throat swab samples were collected from 24 volunteers and spiked 8 each with 1 × 10^5^ copies/mL heat inactivated 2019 novel coronavirus (nCoV; blue diamond), VLPs (red diamond), or water (green diamond). Total RNA was extracted using (**A**) Opentron-2 and (**B**) Eppendorf epMotion. Biological samples were analysed by SYBR one-step RT-qPCR targeting SARS-CoV-2 N gene. Artefact amplification was observed in water-spiked saliva and throat swab samples (green diamond).

Upon demonstrating the efficiency of the miniaturised, TRIzol-based RNA extraction using the semi-automated workflow, the platform was further validated on mock patient samples loaded with SARS-CoV-2 RNA. Saliva and throat swab samples were collected from 24 volunteers and SARS-CoV-2 VLPs and nCoV were separately added into 8 samples each at 1 × 10^5^ copies/ml to simulate clinically relevant concentrations^11^. In the SYBR RT-qPCR assay, up to 3 false positive signals with significant Ct values (Ct < 37) was detected out of the expected 16 positive spiked samples (Fig. 5). Examination of the melt profiles revealed nonconcordant melt peaks, indicating the occurrence of amplification artefacts in both saliva and throat negative control samples. Amplification of nonspecific products in quantitative PCR using SYBR Green-based detection is not uncommon and presents compromises in specificity and reproducibility^12^. Negative detection was observed for the internal control primer set targeted at RNaseP, especially in throat swab samples, in which up to 50% of the 24 samples did not yield signals. This could largely be due to the variability in skill of swab sampling since this was self-administered by untrained volunteers. Non-detection of internal control was noticeably reduced in saliva samples, occurring in 1 to 3 out of 24 samples. These results lend support to the use of saliva as a reliable alternative for detection of SARS-CoV-2 as it alleviates the need for trained personnel for specimen collection and reduces the reliance on swabs and viral transport media.

**Figure 6:**
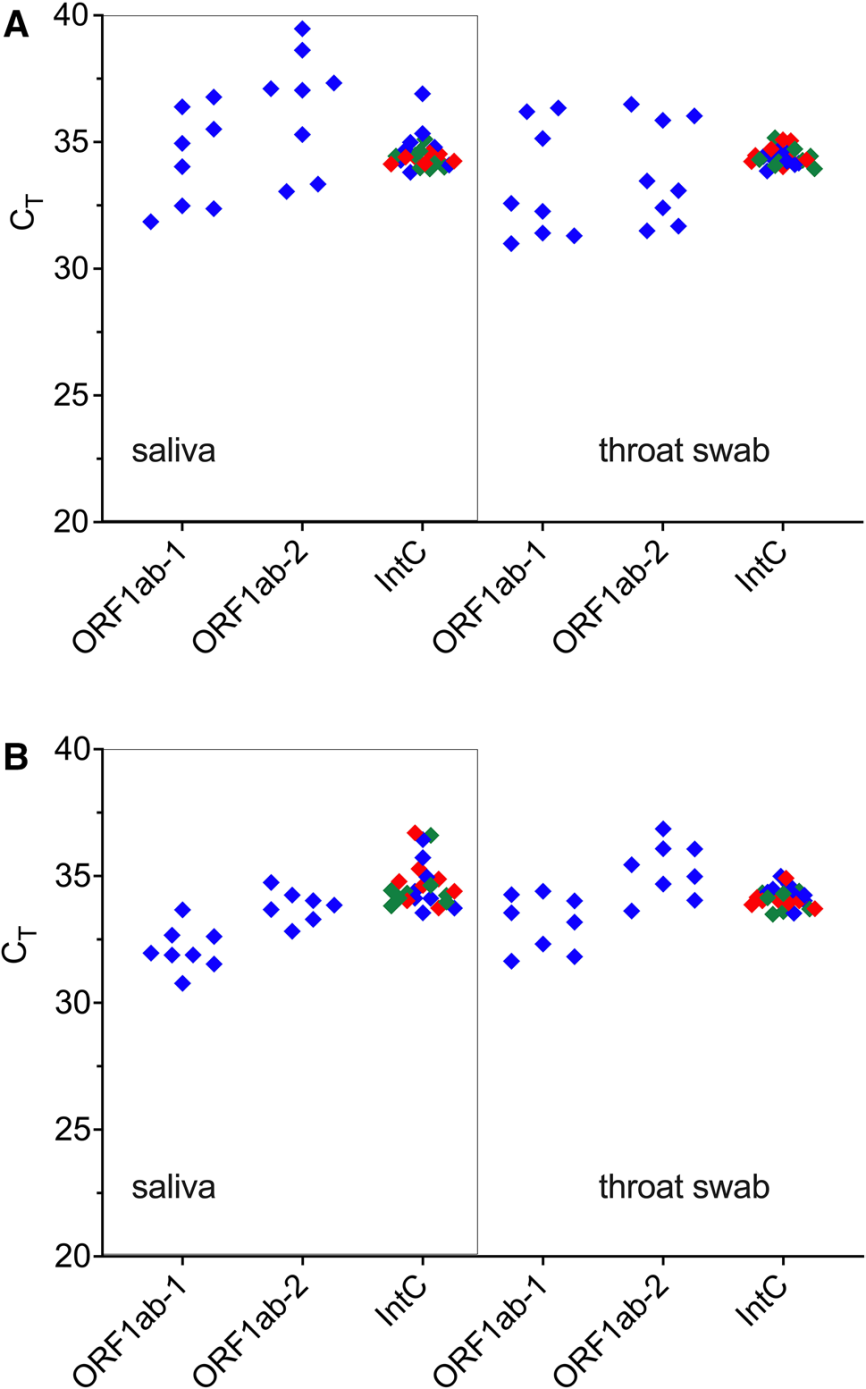
SARS-CoV-2 detection on hypothetical patient samples. Saliva and throat swab samples were collected from 24 volunteers and spiked 8 each with 1 × 10^5^ copies/ml heat inactivated 2019 novel coronavirus (nCoV; blue diamond), VLPs (red diamond), or water (green diamond). Total RNA was extracted using (**A**) Opentron-2 and (**B**) Eppendorf epMotion. Biological samples were analysed by multiplex, probe-based RT-qPCR targeting SARS-CoV-2 ORF1a gene.

To circumvent the challenge of false positives, which puts the diagnostic reliability into question, the analysis was repeated using a multiplex, probe-based RT-qPCR kit deploying three primer–probe sets targeting the ORF1ab region 1 and 2 of the SARS-CoV-2 genome as well as an internal control (Fig. 6). Expectedly, the probe-based assay displayed higher specificity compared to the SYBR Green-based method, producing no false positives in any of the SARS-CoV-2-negative samples. Indeed, all biological samples spiked with VLPs did not generate positive signals as these recombinant VLPs were engineered to carry the SARS-CoV-2 N gene and lacked the ORF1ab region targeted by the RT-qPCR assay used.

**Figure 7:**
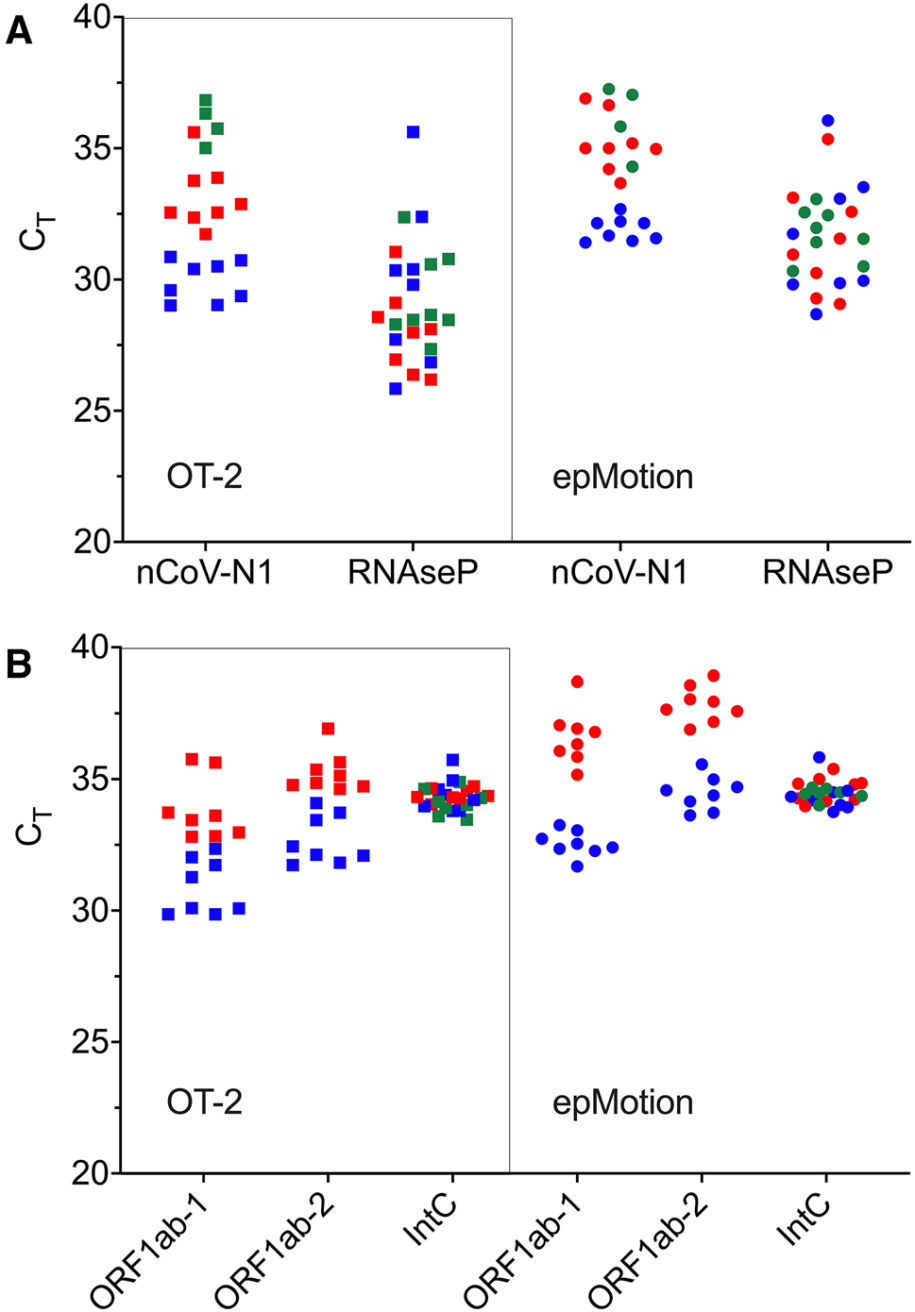
SARS-CoV-2 detection on hypothetical patient samples. Oropharyngeal nasal mid-turbinate samples were collected from 24 volunteers and spiked 8 each with 1 × 10^4^ copies/ml (blue square, blue circle), 1 × 10^5^ copies/ml (red square, red circle), heat inactivated 2019 novel coronavirus (nCoV), or water (green square, green circle). Total RNA was extracted using Opentron-2 (OT-2; *squares*) and Eppendorf epMotion (epMotion; *circles*). (**A**) Biological samples were analysed by SYBR one-step RT-qPCR targeting SARS-CoV-2 N gene. Artefact amplification was observed in water-spiked samples (green square, green circle). (**B**) Biological samples were analysed by multiplex, probe-based RT-qPCR targeting SARS-CoV-2 ORF1a gene.

In a bid to verify if the partial detection of internal control was indeed the result of inconsistencies in self-sampling by untrained volunteers, sampling was repeated by a medically trained personnel familiar with oropharyngeal nasal mid-turbinate swabbing performed in routine Covid-19 testing. This time, detection for the RNaseP internal control in the SYBR RT-qPCR assay was positive for all 24 volunteers (Fig. 7A). The result suggests that the RNA extraction platform is reliable in a realistic, diagnostic setting. Instead of VLPs, 8 biological samples were spiked with 1 × 10^5^ copies/ml nCoV and another 8 with a tenfold lower concentration of 1 × 10^4^ copies/ml nCoV. Occurrence of amplification artefacts recurred as observed in 4 random, false positive signals (Ct ≤ 37) in the negative, water-spiked controls, underlining the shortcoming in specificity of SYBR Green-based detection, particularly for diagnostic analyses (Fig. 7A). Analyses by both SYBR Green- and probe-based RT-qPCR gave positive detection in all 16 SARS-CoV-2-spiked samples, with Ct values higher for those spiked with 1 × 10^4^ copies/ml nCoV (Fig. 7A, B).

As exemplified by the recent, unanticipated surge in Covid-19 testing across the world, supply chain issues in commercial RNA extraction kits can present a rate-limiting obstacle to swift and extensive clinical diagnosis. In a bid to ease the reliance on proprietary, high-throughput RNA extraction kits and their dedicated instruments, a semi-automated, high-throughput kit-free workflow was designed with miniaturization and minimal manual operation in mind (Fig. 1). This approach is notably more cost-effective as kit-based RNA isolation is more expensive than TRIzol-based RNA extraction (Table 1). By further lowering the sample volume from hundreds down to less than 50 µl and by processing in the 96-well format, the semi-automated workflow significantly reduces processing time, but, more importantly, lowers the operational cost which can translate to substantial savings for healthcare test facilities. Based on actual prices for all consumables used, including general laboratory plastic wares such as collection tubes, pipette tips and 96-well plates, as well as chemicals and reagents purchased from local vendors, the cost per sample was as low as less than USD 5 to up to USD 15 without taking into consideration the capital cost for instruments. The price disparity is primarily attributable to the broad price range between the affordable universal SYBR-Green dye-based kits and the more specific but costlier probe-based assays. Even in the absence of automated liquid handlers, the 96-well adapted protocol can still be performed manually using an 8-channel pipette at comparable speed, albeit more labour-intensive. It can be argued that emerging diagnostic strategies involving extraction-free direct RT-qPCR detection offers an even more attractive option that will not only drive the cost lower but also greatly reduce time required for sample processing^13–15^. While holding great promise for SARS-CoV-2 detection, its prospective application for medical surveillance of future novel infectious diseases will require re-validation, thereby necessitating the reliance on the assured reliability of conventional, prior RNA isolation, at least in the early stage of routine clinical testing.

**Table 1:** Cost comparison of RNA extraction methods with high-throughput approach. The cost (USD) per sample includes laboratory consumables such as tips and reagents, but excludes the cost of instruments.

Kit-type	Method	Cost/sample
Current Kit-Free RNA extraction	TRIzol-based	3.80
Analytik Jena-innuPREP^8^	Magnetic beads	4.10
BioBasic-MagicMag	Magnetic beads	8.40
MagBio-HighPrep Viral	Magnetic beads	8.40
Qiagen-EZ1 RNA kit	Silica-based	10.10

## Methods

### Sample collection

Pharyngeal, saliva and oropharyngeal nasal mid-turbinate samples were collected from 24 volunteers. Approval was sought from the ethics committee/institutional review board, the National University of Singapore Office of Safety, Health and Environment (NUS OSHE). All experiments were performed in accordance with NUS OSHE guidelines and regulations. Informed consent was obtained from all volunteers.

The pharyngeal, or throat, swab was performed using a sterile polyester swab with a polystyrene shaft (Deltalab, Spain) according to instructions detailed in Won et al. 2020^10^. After collection, the swab was immediately suspended in 400 μl Dulbecco’s Modified Eagle Medium (DMEM, Gibco, USA) and mixed vigorously to transfer the specimen. Saliva collection was carried out by briefly swirling the saliva in the mouth, pooling and spitting into a sterile 5 ml tube. Oropharyngeal nasal mid-turbinate swabs were conducted by a medically trained healthcare worker using a sterile polyester swab with a polystyrene shaft (Deltalab, Spain) which was suspended directly in 400 μl Dulbecco’s Modified Eagle Medium (DMEM, Gibco, USA) and mixed vigorously to transfer the specimen. Consumption of food, water and brushing of teeth was avoided at least an hour before sampling. All samples were kept on ice until RNA extraction on the same day.

Positive controls for SARS-CoV-2 were prepared using MS2 bacteriophage SARS-CoV-2 virus-like-particles (VLPs, 1700 copies/μl) provided by Crone et al. 2020^8^ and heat-inactivated 2019 novel coronavirus (nCoV, 3.75 × 10^5^ genome copies/μl, VR-1986HK, ATCC) with serial dilutions made using diethylpyrocarbonate (DEPC)-treated water as diluent. For mock SARS-CoV-2 positive samples in the 24 healthy volunteers, both saliva and throat samples from 8 volunteers were spiked with VLPs and another 8 volunteers with nCoV at 1 × 10^5^ copies/ml concentration before RNA extraction. Separately, the oropharyngeal nasal mid-turbinate samples were spiked with 1 × 10^4^ and 1 × 10^5^ copies/ml nCoV for 8 volunteers each.

### Total RNA extraction

Total RNA was extracted from throat, saliva and oropharyngeal nasal mid-turbinate samples using TRIzol (Thermo Fisher Scientific, USA) with modifications optimised for a 96-well format ready for multi-channel pipette or automated liquid handlers. Automated pipetting was performed using an 8-channel pipetting arm of the OT-2 lab robot (OpenTrons, USA) and epMotion 5075 (Eppendorf, Germany). For each extraction, 45 μl of sample was aliquoted into a 96-well PCR plate and 105 μl of TRIzol was added and mixed vigorously by pipetting up-and-down 20 times. After incubation for 10 min at room temperature, 60 μl chloroform was added and mixed vigorously by pipetting 20 times before incubating for another 3 min. After centrifugation on a plate rotor (Eppendorf 5810R, Germany) at a maximum speed of 3200 × *g* for 15 min at 4 °C, 80 μl of the clear, upper aqueous layer was aspirated, transferred to a clean well containing 3 μl glycogen (2 μg/μl) and mixed slowly by pipetting 5 times. An equal volume, or 80 μl, of isopropanol was added next, mixed slowly by pipetting 20 times and allowed to incubate for 10 min at room temperature before centrifugation at 3200 × *g* at 4 °C for 20 min. The supernatant was aspirated and the remaining pellet washed with 150 μl of 75% ethanol by slowly pipetting 5 times before centrifugation again at 3200 × *g* at 4 °C for 10 min. The washing step was carried out twice and the pellet dried at 65 °C for 5 min on a heating block. To solubilise the RNA pellet, 10 μl of DEPC-treated water pre-warmed to 65 °C was added and mixed slowly by pipetting 20 times. The RNA suspension was allowed to incubate for 10 min at room temperature to further improve solubility before use in PCR analysis. The RNA concentration was determined with a microplate reader (BioTek, USA) using the Quant-iT RNA assay kit (Thermo Fisher Scientific, USA).

RNA purification by commercial kits, Qiagen QIAamp Viral RNA kit and RNeasy Mini kit, was carried out according to manufacturer’s instructions using the recommended starting sample volume and final elution volume. The volume of the RNA elute was brought down to a standardised volume of 10 ul by vacuum centrifugation before RT-qPCR analysis.

### Quantitative reverse transcription PCR (RT-qPCR)

For SYBR Green-based detection, RT-qPCR was carried out using Luna Universal One-Step RT-qPCR Kit (New England Biolabs, USA), iTaq Universal SYBR Green One-Step Kit (Bio Rad, USA) and KAPA SYBR FAST One-Step qRT-PCR Master Mix Kit (Kapa Biosystems, USA). In the reaction setup for all kits, 1 μl of extracted RNA was added to half the standard reaction, i.e. 10 μl, volume. The following primer sets were used: human RNase P (5′- CGGTGTTTGCAGATTTGGAC, 5′- CGATATGATTGATAGCAACAACTGAATAGC), US CDC SARS-CoV-2 N1 (5′- GACCCCAAAATCAGCGAAAT, 5′- TCTGGTTACTGCCAGTTGAATCTG). RT-qPCR was performed following manufacturer’s thermal cycling protocol using the Bio Rad CFX96 Real-Time PCR Detection System (Bio Rad, USA) with the threshold setting determined by the CFX Maestro software. In addition, qualitative detection specific for SARS-CoV-2 was conducted using the MiRXES Fortitude Kit 2.0 RUO (MiRXES, Singapore), which is a one-step, multiplex real-time RT-PCR kit with three primer–probe sets targeting the ORF1ab region 1 and 2 of the SARS-CoV-2 genome as well as an internal control. The PCR reaction setup and run profile was carried out according to manufacturer’s instruction with the recommended threshold setting at 50.

All data generated or analysed during this study are included in this published article (and its Supplementary Information file).

## Supplementary Information

Supplementary Information.
