# Supplementary material for: A high-throughput pipeline for scalable kit-free RNA extraction
Source: Sci Rep. 2021 Dec 1;11:23260. doi: 10.1038/s41598-021-02742-w (PMC8636496; doi:10.1038/s41598-021-02742-w)
Supplement: Supplementary file 1 — Supplementary Information. [file 41598_2021_2742_MOESM1_ESM.pdf]

SUPPLEMENTARY INFORMATION

A High-Throughput Pipeline for Scalable Kit-Free RNA Extraction

Ping Han[†,§,‡,#], Maybelle K. Go[†,§,‡,#], Jeng Yeong Chow[†,§,‡,#], Bo Xue[†,§,‡,#], Yan Ping Lim[†,§,‡], Michael A. Crone[*,⊥,^], Marko Storch[*,⊥], Paul S. Freemont[*,⊥,^], Wen Shan Yew[†,§,‡]

[†] Synthetic Biology for Clinical and Technological Innovation, National University of Singapore, 28 Medical Drive, Singapore 117456

[§] Synthetic Biology Translational Research Programme, Yong Loo Lin School of Medicine, National University of Singapore, 14 Medical Drive, Singapore 117599

[‡] Department of Biochemistry, Yong Loo Lin School of Medicine, National University of Singapore, 8 Medical Drive, Singapore 117597

* Section of Structural and Synthetic Biology, Department of Infectious Disease, Faculty of Medicine, Imperial College London, Exhibition road, South Kensington, London SW7 2AZ, UK.

[⊥] London Biofoundry, Translation and Innovation Hub, Imperial College White City Campus, London W12 0BZ, UK.

[^] UK Dementia Research Institute Centre for Care Research and Technology, based at Imperial College London and the University of Surrey.

[#] Authors contributed equally to the work.

Correspondence and requests for materials should be addressed to Wen Shan Yew.

(email: wenshanyew@nus.edu.sg)

Trizol-Based RNA Extraction Protocol: Opentrons OT-2

Opentrons import protocol_api

```
def run(protocol: protocol_api.ProtocolContext):

	column_number = 12	#column_number defines the number of columns on the 96-well plate with samples, starting from column 1, set 12 for a full plate
	discard_tips = 'yes'	#for troubleshooting; yes = tips will be discarded into trash bin, no = tips will be returned to the pipette box

	def left_tips(tip, rack):
		if tip == 'yes':
			left_pipette.drop_tip()
		if tip == 'no':
			left_pipette.return_tip(rack)
	def right_tips(tip, rack):
		if tip == 'yes':
			right_pipette.drop_tip()
		if tip == 'no':
			right_pipette.return_tip(rack)

	m300rack_01 = protocol.load_labware('opentrons_96_tiprack_300ul', '1')
	m300rack_02 = protocol.load_labware('opentrons_96_tiprack_300ul', '4')
	m300rack_03 = protocol.load_labware('opentrons_96_tiprack_300ul', '7')
	m300rack_04 = protocol.load_labware('opentrons_96_tiprack_300ul', '10')
	m300rack_05 = protocol.load_labware('opentrons_96_tiprack_300ul', '11')
	m300rack_06 = protocol.load_labware('opentrons_96_tiprack_300ul', '8')
	m10rack_01 = protocol.load_labware('opentrons_96_tiprack_10ul', '9')
	microplate96_01 = protocol.load_labware('opentrons_96_aluminumblock_generic_pcr_strip_200ul', '2')
	microplate96_02 = protocol.load_labware('opentrons_96_aluminumblock_generic_pcr_strip_200ul', '3')
	reservoir_01 = protocol.load_labware('usascientific_12_reservoir_22ml', '5')
	reservoir_02 = protocol.load_labware('axygen_1_reservoir_250000ul', '6')

	left_pipette = protocol.load_instrument('p300_multi', 'left', tip_racks=[m300rack_01, m300rack_01,
		m300rack_01, m300rack_01, m300rack_01])
	right_pipette = protocol.load_instrument('p10_multi', 'right', tip_racks=[m10rack_01])
```

```
# Add Trizol
for i in range(column_number):
        left_pipette.pick_up_tip(m300rack_01['A'+str(i+1)])
        left_pipette.transfer(105, reservoir_01.wells()[0].bottom(5),
        microplate96_01.wells()[i*8].bottom(5), mix_after=(10, 75),
         new_tip='never')
        left_tips(discard_tips, m300rack_01['A'+str(i+1)])

protocol.delay(seconds=5)
protocol.pause('Incubate Sample!')

# Add chloroform
for i in range(column_number):
        left_pipette.pick_up_tip(m300rack_02['A'+str(i+1)])
        left_pipette.transfer(60, reservoir_01.wells()[2].bottom(5),
        microplate96_01.wells()[i*8].bottom(5), mix_before=(3, 150),
        mix_after=(10, 105), new_tip='never', air_gap=25)
        left_tips(discard_tips, m300rack_02['A'+str(i+1)])

protocol.delay(seconds=5)
protocol.pause('Centrifuge Sample and Replace tips at 1!')

# Transfer upper aqueous phase and aliquot isopropanol
for i in range(column_number):
        left_pipette.pick_up_tip(m300rack_03['A'+str(i+1)])
        left_pipette.flow_rate.aspirate=15
        left_pipette.flow_rate.dispense=100
        left_pipette.aspirate(80, microplate96_01.wells()[(i)*8].bottom(11)) #11.0mm from bottom
        left_pipette.flow_rate.aspirate=100
        left_pipette.dispense(100, microplate96_02.wells()[(i)*8].bottom(5))
        left_pipette.mix(5, 40)
        left_tips(discard_tips, m300rack_03['A'+str(i+1)])
        left_pipette.pick_up_tip(m300rack_01['A'+str(i+1)])
        left_pipette.transfer(80, reservoir_01.wells()[4].bottom(5),
        microplate96_02.wells()[i*8].bottom(5), mix_before=(3, 150),
        mix_after=(20, 100), new_tip='never', air_gap=25)
        left_tips(discard_tips, m300rack_01['A'+str(i+1)])

protocol.delay(seconds=5)
protocol.pause('Centrifuge Sample!')


# Remove supernatant
left_pipette.flow_rate.aspirate=30
left_pipette.flow_rate.dispense=200
```

```
for i in range(column_number):
        left_pipette.pick_up_tip(m300rack_04['A'+str(i+1)])
        left_pipette.transfer(200, microplate96_02.wells()[i*8].bottom(1),
        reservoir_02.wells()[0].bottom(10),
        new_tip='never')
        left_tips(discard_tips, m300rack_04['A'+str(i+1)])
        left_pipette.flow_rate.aspirate=150


# Add 75% ethanol
for i in range(column_number):
        left_pipette.pick_up_tip(m300rack_05['A'+str(i+1)])
        if i < 6:
        left_pipette.transfer(240, reservoir_01.wells()[6].bottom(5),
        microplate96_02.wells()[i*8].bottom(5),
        mix_before=(3, 150), mix_after=(3, 125), new_tip='never', air_gap=25)
        else:
        left_pipette.transfer(240, reservoir_01.wells()[8].bottom(5),
        microplate96_02.wells()[i*8].bottom(5),
        mix_before=(3, 150), mix_after=(3, 125), new_tip='never', air_gap=25)
        left_tips(discard_tips, m300rack_05['A'+str(i+1)])

protocol.delay(seconds=5)
protocol.pause('Centrifuge Sample!')

# Remove 75% ethanol
left_pipette.flow_rate.aspirate=30

for i in range(column_number):
        left_pipette.pick_up_tip(m300rack_06['A'+str(i+1)])
        left_pipette.transfer(260, microplate96_02.wells()[i*8].bottom(1),
        reservoir_02.wells()[0].bottom(10),
        new_tip='never')
        left_tips(discard_tips, m300rack_06['A'+str(i+1)])
        left_pipette.flow_rate.aspirate=150

protocol.pause('Dry Sample!')

# Add water
for i in range(column_number):
        right_pipette.pick_up_tip(m10rack_01['A'+str(i+1)])
        right_pipette.transfer(10, reservoir_01.wells()[10].bottom(5),
        microplate96_02.wells()[i*8].bottom(1),
        mix_after=(3, 5), new_tip='never')
        right_tips(discard_tips, m10rack_01['A'+str(i+1)])
```

Trizol-Based RNA Extraction Protocol: Eppendorf EpMotion

Eppendorf AG
Barkhausenweg 1
22339 Hamburg
Germany

**Application**	1 of 3
**Path**	
**Created**	
**Modified**	
**Software version**	40.7.1.14
**Comment**	Trizol-Based RNA Extraction Protocol

Worktable

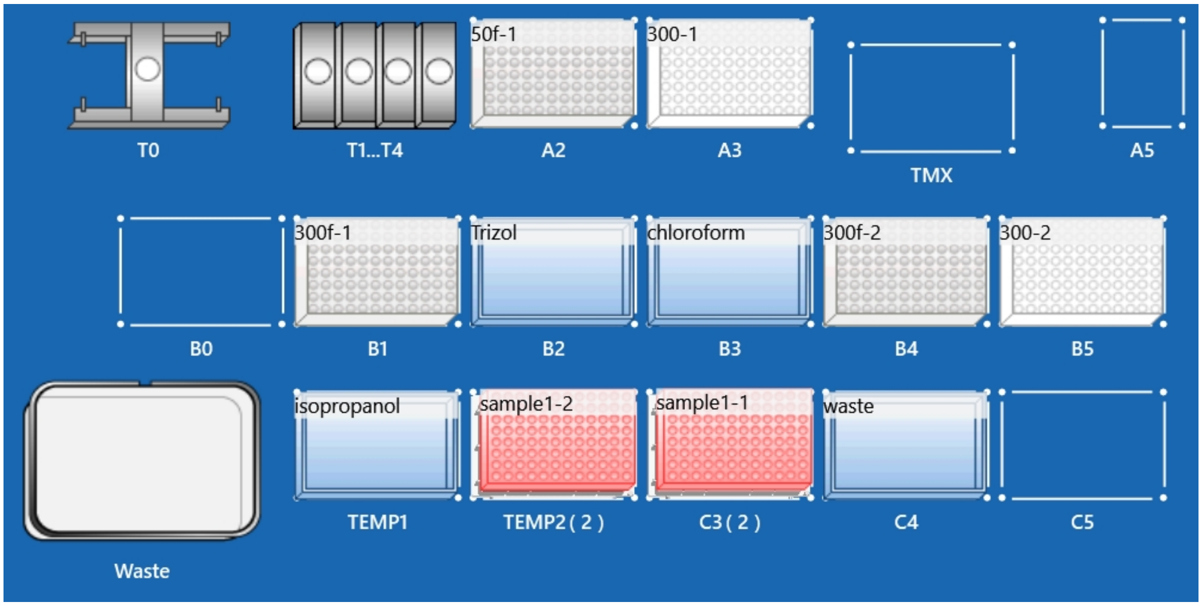

Eppendorf AG
Barkhausenweg 1
22339 Hamburg
Germany

Method

Command 1	Tempering
Location:	TEMP1
Temperature On:	On
Keep temperature:	Off
Temperature:	4 °C

Command 2	Tempering
Location:	TEMP2
Temperature On:	On
Keep temperature:	Off
Temperature:	4 °C

Command 3	Reagent Transfer
Pipette tool:	TM_300_8
Filter Tips:	Yes
Volume:	105 µl
Transfer type:	Pipette
Source:	Trizol
Destination:	sample1-1
Liquid type:	Water
Dosing parameter:	
|-- Speed Aspiration:	11 mm/sec
|-- Speed Dispense:	15.4 mm/sec
|-- Delay Blow:	0 ms

|-- Speed Blow:	10 mm/sec
|-- Movement Blow:	0 % of max movement
|-- Initial stroke:	100 % of max stroke
|-- Prewetting:	0 Cycle(s)
|-- Aspiration liquid offset	3 mm
|-- Dispense liquid offset	-3 mm
Change tips	before each aspiration
Change tips after	0 aspirations
Dip tip after dispensing	50 mm above liquid
Mix before	No
Mix after	Yes
|-- No. of cycles:	20
|-- Speed:	10 mm/sec
|-- Volume:	145 µl
|-- Fixed height:	Yes
|-- Asp./Disp.:	1.2 / 10 mm
Rinse	No
Special	Aspirate from bottom
	Dispense from top

Eppendorf AG
Barkhausenweg 1
22339 Hamburg
Germany

Pattern

**Trizol**		**sample1-1**
1	---->	A1, B1, C1, D1, E1, F1, G1, H1 A2, B2, C2, D2, E2, F2, G2, H2 A3, B3, C3, D3, E3, F3, G3, H3 A4, B4, C4, D4, E4, F4, G4, H4 A5, B5, C5, D5, E5, F5, G5, H5 A6, B6, C6, D6, E6, F6, G6, H6 A7, B7, C7, D7, E7, F7, G7, H7 A8, B8, C8, D8, E8, F8, G8, H8 A9, B9, C9, D9, E9, F9, G9, H9 A10, B10, C10, D10, E10, F10, G10, H10 A11, B11, C11, D11, E11, F11, G11, H11 A12, B12, C12, D12, E12, F12, G12, H12

**Command 4**	**Wait**
Wait Time:	5min. 0sec.
Wait for temperature:	No

**Command 5**	**UserIntervention**
Comment:	
Alarm	No

Eppendorf AG
Barkhausenweg 1
22339 Hamburg
Germany

Command 6	Reagent Transfer
Pipette tool:	TM_300_8
Filter Tips:	No
Volume:	90 µl
Transfer type:	Pipette
Source:	chloroform
Destination:	sample1-1
Liquid type:	Alcohol 98%
Dosing parameter:	
|-- Speed Aspiration:	5 mm/sec
|-- Speed Dispense:	4 mm/sec
|-- Delay Blow:	10 ms
|-- Speed Blow:	0.1 mm/sec
|-- Movement Blow:	0 % of max movement
|-- Initial stroke:	0 % of max stroke
|-- Prewetting:	1 Cycle(s)
|-- Aspiration liquid offset	3 mm
|-- Dispense liquid offset	-20 mm
Change tips	before each aspiration
Change tips after	0 aspirations
Dip tip after dispensing	30 mm above liquid
Mix before	No
Mix after	Yes
|-- No. of cycles:	20
|-- Speed:	10 mm/sec
|-- Volume:	235 µl
|-- Fixed height:	Yes

|-- Asp./Disp.:	1.2 / 10 mm
Rinse	No
Special	Aspirate from bottom
	Dispense from top
Pattern	

**chloroform**		**sample1-1**
1	---->	A1, B1, C1, D1, E1, F1, G1, H1 A2, B2, C2, D2, E2, F2, G2, H2 A3, B3, C3, D3, E3, F3, G3, H3 A4, B4, C4, D4, E4, F4, G4, H4 A5, B5, C5, D5, E5, F5, G5, H5 A6, B6, C6, D6, E6, F6, G6, H6 A7, B7, C7, D7, E7, F7, G7, H7 A8, B8, C8, D8, E8, F8, G8, H8 A9, B9, C9, D9, E9, F9, G9, H9 A10, B10, C10, D10, E10, F10, G10, H10 A11, B11, C11, D11, E11, F11, G11, H11 A12, B12, C12, D12, E12, F12, G12, H12

**Command 7**	**Wait**
Wait Time:	3min. 0sec.
Wait for temperature:	No

Command 8	UserIntervention
Comment:	Centrifugation at max speed; 15 min
Alarm	No

Command 9	Sample transfer
Pipette tool:	TM_300_8
Filter Tips:	Yes
Volume:	60 µl
Transfer type:	Pipette
Source:	sample1-1
Destination:	sample1-2
Liquid type:	Water
Dosing parameter:	
|-- Speed Aspiration:	2 mm/sec
|-- Speed Dispense:	8 mm/sec
|-- Delay Blow:	10 ms
|-- Speed Blow:	33 mm/sec
|-- Movement Blow:	0 % of max movement
|-- Initial stroke:	100 % of max stroke
|-- Prewetting:	0 Cycle(s)
|-- Aspiration liquid offset	0 mm
|-- Dispense liquid offset	-3 mm
Change tips	before aspirating a new sample
Change tips after	0 aspirations
Dip tip after dispensing	50 mm above liquid
Mix before	No
Mix after	No

Rinse	No
Special	Aspirate 9.2 mm above well bottom without tip movement
	Dispense from top
Pattern	

**sample1-1**		**sample1-2**
A1, B1, C1, D1, E1, F1, G1, H1	---->	A1, B1, C1, D1, E1, F1, G1, H1
A2, B2, C2, D2, E2, F2, G2, H2	---->	A2, B2, C2, D2, E2, F2, G2, H2
A3, B3, C3, D3, E3, F3, G3, H3	---->	A3, B3, C3, D3, E3, F3, G3, H3
A4, B4, C4, D4, E4, F4, G4, H4	---->	A4, B4, C4, D4, E4, F4, G4, H4
A5, B5, C5, D5, E5, F5, G5, H5	---->	A5, B5, C5, D5, E5, F5, G5, H5
A6, B6, C6, D6, E6, F6, G6, H6	---->	A6, B6, C6, D6, E6, F6, G6, H6
A7, B7, C7, D7, E7, F7, G7, H7	---->	A7, B7, C7, D7, E7, F7, G7, H7
A8, B8, C8, D8, E8, F8, G8, H8	---->	A8, B8, C8, D8, E8, F8, G8, H8
A9, B9, C9, D9, E9, F9, G9, H9	---->	A9, B9, C9, D9, E9, F9, G9, H9
A10, B10, C10, D10, E10, F10, G10, H10	---->	A10, B10, C10, D10, E10, F10, G10, H10
A11, B11, C11, D11, E11, F11, G11, H11	---->	A11, B11, C11, D11, E11, F11, G11, H11
A12, B12, C12, D12, E12, F12, G12, H12	---->	A12, B12, C12, D12, E12, F12, G12, H12

**Command 10**	**Reagent Transfer**
Pipette tool:	TM_300_8
Filter Tips:	No
Volume:	90 µl

Transfer type:	Pipette
Source:	isopropanol
Destination:	sample1-2
Liquid type:	Alcohol 98%
Dosing parameter:	
|-- Speed Aspiration:	2.2 mm/sec
|-- Speed Dispense:	2.2 mm/sec
|-- Delay Blow:	0 ms
|-- Speed Blow:	0.2 mm/sec
|-- Movement Blow:	0 % of max movement
|-- Initial stroke:	100 % of max stroke
|-- Prewetting:	0 Cycle(s)
|-- Aspiration liquid offset	3 mm
|-- Dispense liquid offset	-3 mm
Change tips	before each aspiration
Change tips after	0 aspirations
Dip tip after dispensing	50 mm above liquid
Mix before	No
Mix after	Yes
|-- No. of cycles:	5
|-- Speed:	8 mm/sec
|-- Volume:	158 µl
|-- Fixed height:	Yes
|-- Asp./Disp.:	1.2 / 20 mm
Rinse	No
Special	Aspirate from bottom
	Dispense from top

Pattern

isopropanol		sample1-2
1	---->	A1, B1, C1, D1, E1, F1, G1, H1 A2, B2, C2, D2, E2, F2, G2, H2 A3, B3, C3, D3, E3, F3, G3, H3 A4, B4, C4, D4, E4, F4, G4, H4 A5, B5, C5, D5, E5, F5, G5, H5 A6, B6, C6, D6, E6, F6, G6, H6 A7, B7, C7, D7, E7, F7, G7, H7 A8, B8, C8, D8, E8, F8, G8, H8 A9, B9, C9, D9, E9, F9, G9, H9 A10, B10, C10, D10, E10, F10, G10, H10 A11, B11, C11, D11, E11, F11, G11, H11 A12, B12, C12, D12, E12, F12, G12, H12

**Command 11**	**Wait**
Wait Time:	10min. 0sec.
Wait for temperature:	Yes
Location:	TEMP2

**Command 12**	**UserIntervention**
Comment:	Centrifugation at max speed; 20 min

Alarm	No

Command 13	Empty vessel
Empty tool:	TM_50_8
Filter Tips:	Yes
Volume:	50 µl
Offset:	1 mm
Delay:	0 ms
Repeat:	1
Empty tool positions [mm]:	1. Positon { X: 0, Y: 0 }
Pipette tool:	TM_50_8
Filter Tips:	Yes
Source:	sample1-2
Destination:	waste
Liquid type (Empty tool):	Alcohol 98%
Dosing parameter:	
|-- Speed Aspiration:	3 mm/sec
|-- Speed Dispense:	25 mm/sec
|-- Delay Blow:	0 ms
|-- Speed Blow:	5 mm/sec
|-- Movement Blow:	0 % of max movement
|-- Initial stroke:	100 % of max stroke
|-- Prewetting:	0 Cycle(s)
|-- Aspiration liquid offset	3 mm
|-- Dispense liquid offset	-3 mm
Liquid type (Pipette tool):	Water
Dosing parameter:	default
Change tips	before each aspiration

Eppendorf AG
Barkhausenweg 1
22339 Hamburg
Germany

Change tips after	0 aspirations
Dip tip after dispensing	50 mm above liquid
Special	Dispense from top
Pattern	

**sample1-2**		**waste**
A1, B1, C1, D1, E1, F1, G1, H1 A2, B2, C2, D2, E2, F2, G2, H2 A3, B3, C3, D3, E3, F3, G3, H3 A4, B4, C4, D4, E4, F4, G4, H4 A5, B5, C5, D5, E5, F5, G5, H5 A6, B6, C6, D6, E6, F6, G6, H6 A7, B7, C7, D7, E7, F7, G7, H7 A8, B8, C8, D8, E8, F8, G8, H8 A9, B9, C9, D9, E9, F9, G9, H9 A10, B10, C10, D10, E10, F10, G10, H10 A11, B11, C11, D11, E11, F11, G11, H11 A12, B12, C12, D12, E12, F12, G12, H12	---->	1

Eppendorf AG
Barkhausenweg 1
22339 Hamburg
Germany

**Application**	2 of 3
**Path**	
**Created**	
**Modified**	
**Software version**	40.7.1.14
**Comment**	Trizol-Based RNA Extraction Protocol

Worktable

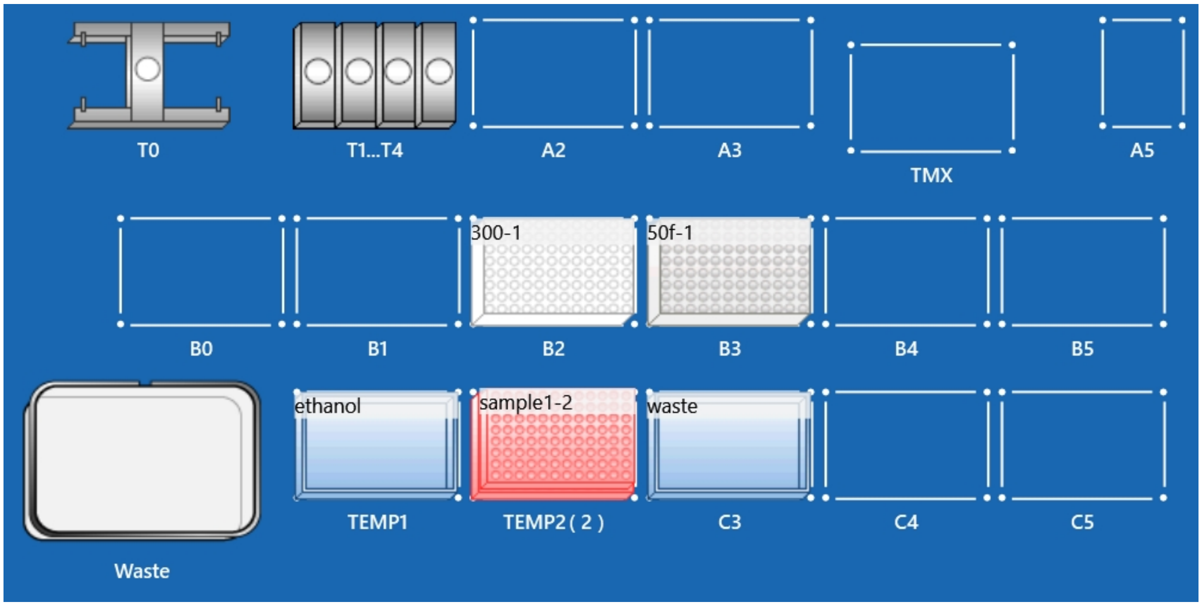

Eppendorf AG
Barkhausenweg 1
22339 Hamburg
Germany

Method

Command 1	Reagent Transfer
Pipette tool:	TM_300_8
Filter Tips:	No
Volume:	150 µl
Transfer type:	Pipette
Source:	ethanol
Destination:	sample1-2
Liquid type:	Alcohol 75%
Dosing parameter:	
|-- Speed Aspiration:	6.6 mm/sec
|-- Speed Dispense:	6.6 mm/sec
|-- Delay Blow:	30 ms
|-- Speed Blow:	4 mm/sec
|-- Movement Blow:	0 % of max movement
|-- Initial stroke:	100 % of max stroke
|-- Prewetting:	1 Cycle(s)
|-- Aspiration liquid offset	3 mm
|-- Dispense liquid offset	-5 mm
Change tips	before each aspiration
Change tips after	0 aspirations
Dip tip after dispensing	50 mm above liquid
Mix before	No
Mix after	Yes
|-- No. of cycles:	5

|-- Speed:	8 mm/sec
|-- Volume:	158 µl
|-- Fixed height:	Yes
|-- Asp./Disp.:	1.2 / 20 mm
Rinse	No
Special	Aspirate from bottom
	Dispense from top
Pattern	

**ethanol**		**sample1-2**
1	---->	A1, B1, C1, D1, E1, F1, G1, H1 A2, B2, C2, D2, E2, F2, G2, H2 A3, B3, C3, D3, E3, F3, G3, H3 A4, B4, C4, D4, E4, F4, G4, H4 A5, B5, C5, D5, E5, F5, G5, H5 A6, B6, C6, D6, E6, F6, G6, H6 A7, B7, C7, D7, E7, F7, G7, H7 A8, B8, C8, D8, E8, F8, G8, H8 A9, B9, C9, D9, E9, F9, G9, H9 A10, B10, C10, D10, E10, F10, G10, H10 A11, B11, C11, D11, E11, F11, G11, H11 A12, B12, C12, D12, E12, F12, G12, H12

**Command 2**	**UserIntervention**

Eppendorf AG
Barkhausenweg 1
22339 Hamburg
Germany

Comment:	Centrifugation at max speed; 10 min
Alarm	No

**Command 3**	**Empty vessel**
Empty tool:	TM_50_8
Filter Tips:	Yes
Volume:	50 µl
Offset:	1 mm
Delay:	0 ms
Repeat:	1
Empty tool positions [mm]:	1. Positon { X: 0, Y: 0 }
Pipette tool:	TM_50_8
Filter Tips:	Yes
Source:	sample1-2
Destination:	waste
Liquid type (Empty tool):	Alcohol 98%
Dosing parameter:	
|-- Speed Aspiration:	3 mm/sec
|-- Speed Dispense:	17.6 mm/sec
|-- Delay Blow:	0 ms
|-- Speed Blow:	1.1 mm/sec
|-- Movement Blow:	0 % of max movement
|-- Initial stroke:	100 % of max stroke
|-- Prewetting:	0 Cycle(s)
|-- Aspiration liquid offset	3 mm
|-- Dispense liquid offset	-3 mm
Liquid type (Pipette tool):	Water
Dosing parameter:	default

Eppendorf AG
Barkhausenweg 1
22339 Hamburg
Germany

Change tips	before each aspiration
Change tips after	0 aspirations
Dip tip after dispensing	50 mm above liquid
Special	Dispense from top
Pattern	

sample1-2		waste
A1, B1, C1, D1, E1, F1, G1, H1 A2, B2, C2, D2, E2, F2, G2, H2 A3, B3, C3, D3, E3, F3, G3, H3 A4, B4, C4, D4, E4, F4, G4, H4 A5, B5, C5, D5, E5, F5, G5, H5 A6, B6, C6, D6, E6, F6, G6, H6 A7, B7, C7, D7, E7, F7, G7, H7 A8, B8, C8, D8, E8, F8, G8, H8 A9, B9, C9, D9, E9, F9, G9, H9 A10, B10, C10, D10, E10, F10, G10, H10 A11, B11, C11, D11, E11, F11, G11, H11 A12, B12, C12, D12, E12, F12, G12, H12	---->	1

Eppendorf AG
Barkhausenweg 1
22339 Hamburg
Germany

**Application**	3 of 3
**Path**	
**Created**	
**Modified**	
**Software version**	40.7.1.14
**Comment**	Trizol-Based RNA Extraction Protocol

Worktable

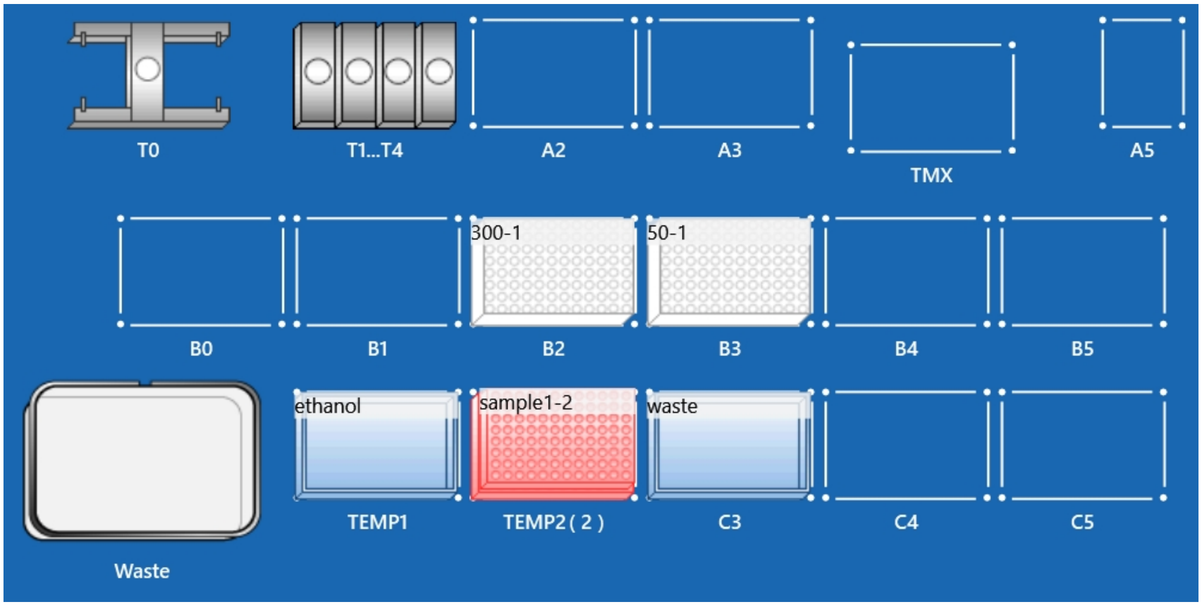

Eppendorf AG
Barkhausenweg 1
22339 Hamburg
Germany

Method

Command 1	Reagent Transfer
Pipette tool:	TM_300_8
Filter Tips:	No
Volume:	150 µl
Transfer type:	Pipette
Source:	ethanol
Destination:	sample1-2
Liquid type:	Alcohol 75%
Dosing parameter:	
|-- Speed Aspiration:	6.6 mm/sec
|-- Speed Dispense:	6.6 mm/sec
|-- Delay Blow:	30 ms
|-- Speed Blow:	4 mm/sec
|-- Movement Blow:	0 % of max movement
|-- Initial stroke:	100 % of max stroke
|-- Prewetting:	1 Cycle(s)
|-- Aspiration liquid offset	3 mm
|-- Dispense liquid offset	-5 mm
Change tips	before each aspiration
Change tips after	0 aspirations
Dip tip after dispensing	50 mm above liquid
Mix before	No
Mix after	Yes
|-- No. of cycles:	5

|-- Speed:	8 mm/sec
|-- Volume:	158 µl
|-- Fixed height:	Yes
|-- Asp./Disp.:	1.2 / 20 mm
Rinse	No
Special	Aspirate from bottom
	Dispense from top
Pattern	

**ethanol**		**sample1-2**
1	---->	A1, B1, C1, D1, E1, F1, G1, H1 A2, B2, C2, D2, E2, F2, G2, H2 A3, B3, C3, D3, E3, F3, G3, H3 A4, B4, C4, D4, E4, F4, G4, H4 A5, B5, C5, D5, E5, F5, G5, H5 A6, B6, C6, D6, E6, F6, G6, H6 A7, B7, C7, D7, E7, F7, G7, H7 A8, B8, C8, D8, E8, F8, G8, H8 A9, B9, C9, D9, E9, F9, G9, H9 A10, B10, C10, D10, E10, F10, G10, H10 A11, B11, C11, D11, E11, F11, G11, H11 A12, B12, C12, D12, E12, F12, G12, H12

**Command 2**	**UserIntervention**

Comment:	Centrifugation at max speed; 10 min
Alarm	No

**Command 3**	**Empty vessel**
Empty tool:	TM_50_8
Filter Tips:	No
Volume:	50 µl
Offset:	1 mm
Delay:	0 ms
Repeat:	2
Empty tool positions [mm]:	1. Positon { X: 0, Y: 0 }
Pipette tool:	TM_50_8
Filter Tips:	No
Source:	sample1-2
Destination:	waste
Liquid type (Empty tool):	Alcohol 98%
Dosing parameter:	
|-- Speed Aspiration:	3 mm/sec
|-- Speed Dispense:	17.6 mm/sec
|-- Delay Blow:	0 ms
|-- Speed Blow:	1.1 mm/sec
|-- Movement Blow:	0 % of max movement
|-- Initial stroke:	100 % of max stroke
|-- Prewetting:	0 Cycle(s)
|-- Aspiration liquid offset	3 mm
|-- Dispense liquid offset	-3 mm
Liquid type (Pipette tool):	Water
Dosing parameter:	default

Change tips	before each aspiration
Change tips after	0 aspirations
Dip tip after dispensing	50 mm above liquid
Special	Dispense from top
Pattern	

**sample1-2**		**waste**
A1, B1, C1, D1, E1, F1, G1, H1 A2, B2, C2, D2, E2, F2, G2, H2 A3, B3, C3, D3, E3, F3, G3, H3 A4, B4, C4, D4, E4, F4, G4, H4 A5, B5, C5, D5, E5, F5, G5, H5 A6, B6, C6, D6, E6, F6, G6, H6 A7, B7, C7, D7, E7, F7, G7, H7 A8, B8, C8, D8, E8, F8, G8, H8 A9, B9, C9, D9, E9, F9, G9, H9 A10, B10, C10, D10, E10, F10, G10, H10 A11, B11, C11, D11, E11, F11, G11, H11 A12, B12, C12, D12, E12, F12, G12, H12	---->	1

**Command 4**	**Tempering**
Location:	TEMP2
Temperature On:	On

Keep temperature:	On
Temperature:	65 °C

**Command 5**	**Wait**
Wait Time:	5min. 0sec.
Wait for temperature:	No
